# Real-world effectiveness of aripiprazole once-monthly REACT study: Pooled analysis of two noninterventional studies

**DOI:** 10.1192/j.eurpsy.2022.27

**Published:** 2022-07-20

**Authors:** Daniel Schöttle, Guerline Clerzius, Wolfgang Janetzky, Oloruntoba Oluboka, Marc-André Roy, François Therrien, Klaus Wiedemann

**Affiliations:** 1 Klinik für Psychiatrie und Psychotherapie, Zentrum für Psychosoziale Medizin, Universitätsklinikum Hamburg-Eppendorf, Hamburg, Germany; 2 Lundbeck Canada Inc., Saint-Laurent, Québec, Canada; 3 Lundbeck GmbH, Hamburg, Germany; 4 University of Calgary, Calgary, Alberta, Canada; 5 Département de Psychiatrie et Neurosciences, Pavillon Ferdinand-Vandry, Local 4889, Université Laval, Québec, Québec, Canada; 6 Centre de Recherche CERVO, Québec, Québec, Canada; 7 Clinique Notre-Dame des Victoires, Québec, Québec, Canada; 8 Otsuka Canada Pharmaceutical Inc., Saint-Laurent, Québec, Canada

**Keywords:** Aripiprazole once-monthly, long-acting injectable, noninterventional study, real-world evidence, schizophrenia

## Abstract

**Background:**

Noninterventional naturalistic studies are an important complement to randomized controlled trials. Aripiprazole once-monthly (AOM) is an atypical antipsychotic in a long-acting injectable formulation.

**Methods:**

A pooled analysis of two noninterventional studies was undertaken to validate previous results on AOM effectiveness and safety in a larger population and improve statistical power for preplanned subgroup analyses. We analyzed data from 409 patients with schizophrenia who were treated with AOM and were enrolled in noninterventional studies in Germany (via noninterventional studies registry 15,960 N) and Canada (NCT02131415). Data collected at baseline, 3 and 6 months were analyzed. Among the endpoints were psychopathology (brief psychiatric rating scale [BPRS]) and disease severity (clinical global impression [CGI]).

**Results:**

Mean patient age was 38.9 (SD 14.8) years, and 59.9% were male. BPRS decreased from 48.1 (SD 15.6) at baseline to 36.5 (SD 13.7) at month 6 (*p* < 0.001). CGI decreased from 4.47 (SD 0.90) at baseline to 3.64 (SD 1.16) at month 6 (*p* < 0.001). A total of 54.4% were responders (at least 20% reduction) on the BPRS, and 56.5% had a CGI-S-score that was at least 1 level better than baseline. A total of 43.4% were considered responders on both the BPRS and CGI scales. A total of 45.2% were considered in remission. Adverse events were rare and corresponded to the previously known safety profile of AOM.

**Conclusions:**

Treatment with AOM for patients with schizophrenia appeared effective and safe under real-life conditions.

## Introduction

Antipsychotic medication is highly effective in the treatment of patients with schizophrenia, lowering the risk of relapse by 2–6 times in first- and multiepisode patients [1–3]. However, nonadherence to medication is often observed in patients with schizophrenia and is related to a significantly increased risk of relapse. Improving adherence and providing a continuous psychopharmacological treatment is a major challenge [4–6].

One way to improve adherence is the use of long-acting injectable antipsychotics (LAI) that offer reliable medication delivery and stable pharmacokinetics and also facilitate monitoring of nonadherence, thereby decreasing, for example, the risk of misuse, of unnecessary medication changes, or of evaluating an insufficient treatment response as “therapy refractive” [7]. There is growing evidence that LAIs should not be seen as a last resort, but rather used in the early phase of the disorder to prevent relapse and hospitalization, as recommended for instance in Québec’s guidelines [8]. In the most recent and comprehensive meta-analysis studying the effectiveness of LAIs across different study designs, LAIs were more effective in preventing hospitalization or relapse in settings ranging from restricted research (randomized controlled trials—RCTs) to real-word application (cohort and pre–post studies) [9]. Long-term advantages of LAIs also include a reduced mortality, not only versus placebo but also versus oral medication [10].

Aripiprazole once-monthly (AOM) is an atypical antipsychotic in a long-acting injectable formulation. AOM has been found in RCTs to be superior to placebo [11] and noninferior to oral aripiprazole [12]. Also, it has been found to be superior to paliperidone palmitate once-monthly in terms of quality of life, especially in patients younger than 35 years [13]. However, RCTs are not readily generalizable to clinical practice due to the exclusion criteria and the somewhat artificial treatment setting [14]. Noninterventional studies are therefore an important complement to RCTs, as they observe patients with many comorbidities who are treated with multiple medications, which is more representative of everyday practice.

Recently, the quasi-naturalistic study PRELAPSE found AOM to be superior to routine care in terms of delaying hospitalizations [15]. PRELAPSE can be considered quasi-naturalistic since treatment centers, not patients, were randomized. In this trial, staff at centers randomized to AOM also received communication trainings in order to improve patient acceptance and adherence.

Also, noninterventional studies in Germany [16] and Canada [17] found that starting AOM as per country-specific labeling led to symptomatic and functional improvements. Here, we pooled and reanalyzed data from these studies in an effort to validate previous results on AOM effectiveness and safety in a larger population and to improve statistical power for preplanned subgroup analyses.

## Methods

This is a post hoc analysis of pooled data from two noninterventional studies on real-life use of AOM in patients with schizophrenia conducted in Germany [16] and Canada [17].

In this analysis, we included all patients that had been treated with AOM and for which at least a baseline and one post-baseline assessment (at month 3 or month 6) was available. Four hundred and nine patients with schizophrenia (at least 18 years old) were included in this analysis. For two patients, no post-baseline assessment was available and they were therefore excluded from the analysis.

In the German trial, outpatients with schizophrenia according to ICD-10 who had been pretreated with oral aripiprazole per German product label were eligible for inclusion after the treating physician had prescribed AOM. Patients were recruited from 75 centers in Germany. The planned observation time for each patient was 6 months. Exclusion criteria for the study were contraindications for AOM, being a member or being related to a member of the study staff, pregnancy, planning a pregnancy, breastfeeding, or expected reluctance to follow the prespecified monitoring plan (as assessed by the treating psychiatrist). Patients with treatment resistant disease or users of clozapine were specifically not excluded. At baseline, all patients had CGI-S-scores of 3 (“mildly ill”) or worse [16]. The treating physician decided on the switch to AOM. Patients were to be switched to AOM as per German product label, that is, patients were to be treated with a fixed dose of oral aripiprazole before the first injection. After the first injection, oral aripiprazole was to be taken concomitantly for 2 weeks and then discontinued.

In the Canadian trial, adult patients (at least 19 years old for patients from British Columbia) with a CGI-S-score of at least 3 (“mildly ill”) were eligible for inclusion after the treating psychiatrist had decided to prescribe AOM. Patients were recruited from 17 Canadian community or hospital-based centers. Exclusion criteria were inability to provide informed consent, contraindications for AOM, having received AOM previously, presenting with significant suicidal risk, pregnancy or lactation. Patients with treatment resistant disease or users of clozapine were specifically not excluded. The planned observation time was 2 years, but the study was terminated earlier after a preplanned interim analysis. Here, data from the first 6 months were analyzed to make pooling with the German study data feasible. Patients were to be switched to AOM as per Canadian product label, that is, patients were treated with oral antipsychotics before the first injection. In patients who had never taken aripiprazole, tolerability had to be established with oral aripiprazole. After the first injection, oral aripiprazole was to be taken concomitantly for 2 weeks and then discontinued.

Brief psychiatric rating scale (BPRS) [18] and clinical global impression–Severity (CGI-S) [19] were among the endpoints of both studies and are here reanalyzed for the pooled data. Data from visits at baseline, month 3 and month 6 were pooled from both studies, as well as adverse events up to month 6. The primary outcome was the change of BPRS total score at month 6 compared to baseline.

The BPRS measures symptom severity on 18 items with scores between 1 (not present) to 7 (extremely severe), yielding total scores between 18 and 126. CGI-S is a measure to report the current global illness state of a patient on a scale from 1 (normal, not at all ill) to 7 (extremely ill). Both were evaluated by the treating psychiatrists on the basis of semi-structured interviews completed during the study visits. The interrater reliability of the BPRS has been reported to range from 0.87 to 0.97, and the interrater reliability of the CGI-S was 0.66 in a small study [20].

We defined a response on the BPRS scale as an improvement of at least 20% versus baseline, and a response on the CGI-S as an improvement of at least one level at study endpoint. Furthermore, we defined remission as a score of 3 or less on the following BPRS items for at least 3 months, that is, two consecutive visits: grandiosity, suspiciousness, unusual thought content, hallucinatory behavior, conceptual disorder, mannerisms, and blunted affect. These criteria are based on the Andreasen criteria [21], but with the time criterion shortened from 3 to 6 months owing to the study duration of only 6 months.

We used the Wilcoxon signed rank test for paired samples, and the Wilcoxon rank sum test for independent samples. Changes in marginal distributions in contingency tables of categorical outcomes were analyzed using Bhapkar’s test, whereas the binomial test was used to analyze proportions within one group of patients. Missing values were imputed using the last observation carried forward (LOCF) method if there was a value for T0 and at least one post-baseline time point. All tests were two-sided with alpha = 0.05, with no correction for multiple testing for secondary outcomes. We also analyzed subgroups of patients≤35 years and >35 years due to the hypothesis that younger patients show a better response, as has been found in the QUALIFY study [13].

## Results

### Demographics

Four-hundred and nine treated across 87 centers were analyzed. Two-hundred and forty patients were included from the German population, and 169 patients from the Canadian population. Mean duration of observation was 5.49 months (SD: 1.43). Three-hundred and eighty-four patients (93.9%) completed the studies until month 6. Reasons for discontinuation during the first 6 months or at month 6 included lack of effectiveness (2.7%), adverse drug reaction (1.7%) or other reason (7.8%). Baseline demographics and clinical characteristics are given in Table 1.

**Table 1.** Baseline demographics and clinical characteristics.

	Total population (*n* = 409)	Patients ≤35 years (*n* = 199)	Patients >35 years (*n* = 210)
Age (years), mean (SD)	38.9 (14.8)	27.0 (4.8)	50.2 (11.9)
Sex male, *n* (%)	245 (59.9)	136 (68.3)	109 (51.9)
Body mass index (kg/m^2^), mean (SD)	29.2 (6.9)	28.2 (6.7)	30.0 (6.9)
Age at diagnosis (years), mean (SD)	29.2 (11.7)	22.7 (4.3)	35.3 (13.2)
Duration of disease since diagnosis (years), mean (SD)	9.8 (10.3)	4.2 (4.4)	15.0 (11.6)

### Dosing

The mean AOM dose at study start was 374 mg (SD: 50.5); 315 patients (77.0%) received a dose of 400 mg. Three-hundred and thirty-six patients (82.2%) received concomitant treatment when starting AOM. For most patients (*n* = 316, 77.3%), this was oral aripiprazole as recommended in the product label. Other concomitant antipsychotics that more than 2% of the patients received were quetiapine (*n* = 26, 6.4%), olanzapine (*n* = 22, 5.4%), clozapine (*n* = 12, 2.9%), and risperidone (*n* = 12, 2.9%). One-hundred and eighty-four patients of the 316 taking concomitant aripiprazole discontinued oral aripiprazole earlier or later than recommended in the product label.

### Symptom severity: BPRS

**Figure 1.**
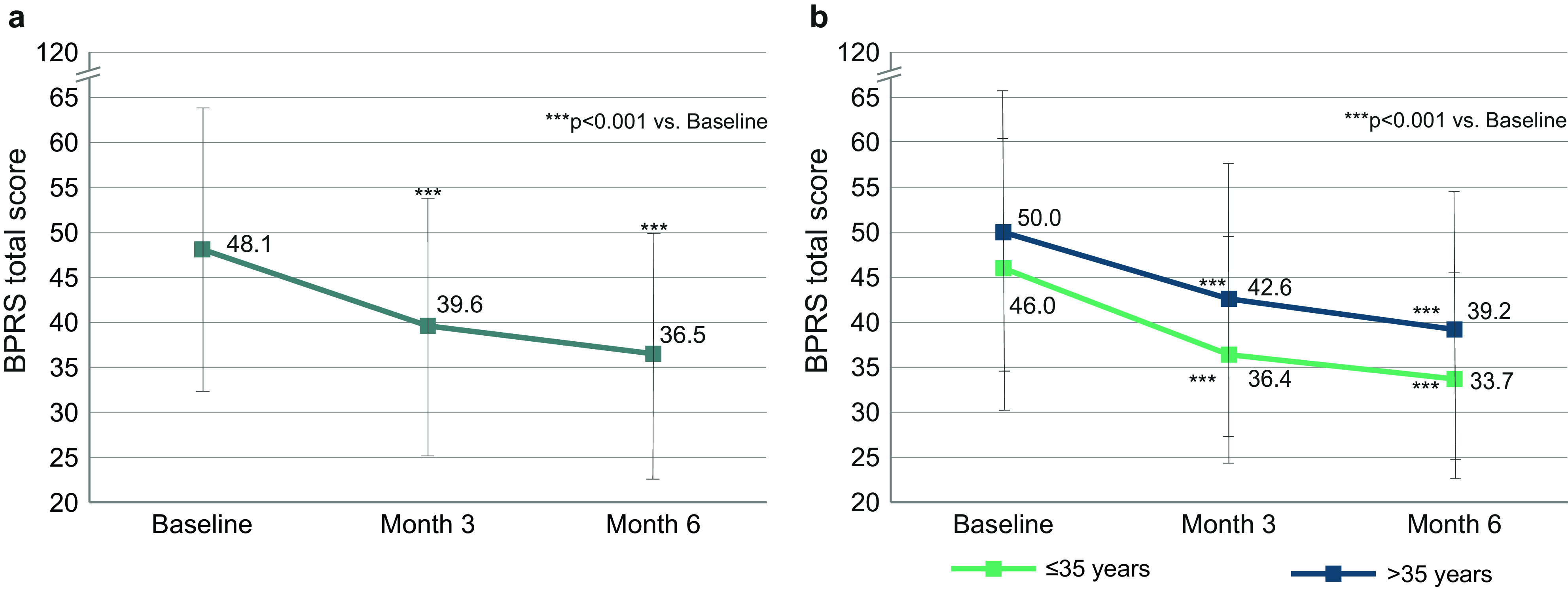
BPRS total score. (A) Change of BPRS total score in the total population (*n* = 395). (B) Change of BPRS total score in patients ≤35 years (*n* = 194) and >35 years (*n* = 201). Error bars represent standard deviations. Missing data were imputed using the last observation carried forward method.

At baseline, the mean BPRS total score was 48.1 (SD 15.6) (Figure 1A). During 6 months of treatment, the mean score improved by 11.6 (SD 14.2) (or 38.5%) to reach 36.5 (SD 13.7) at month 6. Compared to baseline, improvements at month 3 and month 6 were statistically significant (*p* < 0.001). Younger patients ≤35 years had less severe symptoms at baseline and throughout the first 6 months of treatment compared to older patients (Figure 1B). In younger patients, the improvement was −12.4 points during 6 months, which translates to a 44.3% improvement (taking into account the minimal BPRS rating of 18 points); in older patients it was −10.8 points (33.8%).

### Response on the BPRS

**Figure 2.**
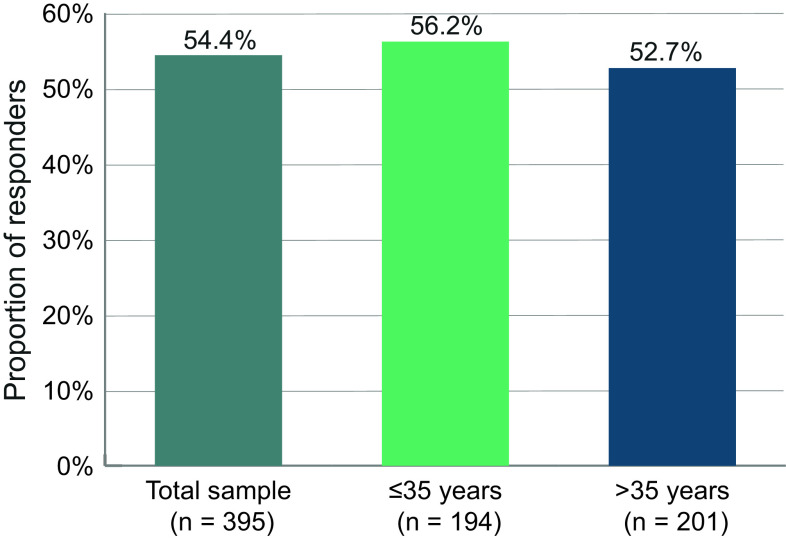
Proportion of responders. Patients were considered responders if they showed an improvement of at least 20% of the BPRS total score.

A total of 54.4% of the patients achieved a response on the BPRS, defined as an improvement of at least 20% versus baseline, within 6 months (Figure 2). The proportion was 56.2% for patients ≤35 years and 52.7% for older patients.

### Remission

**Figure 3.**
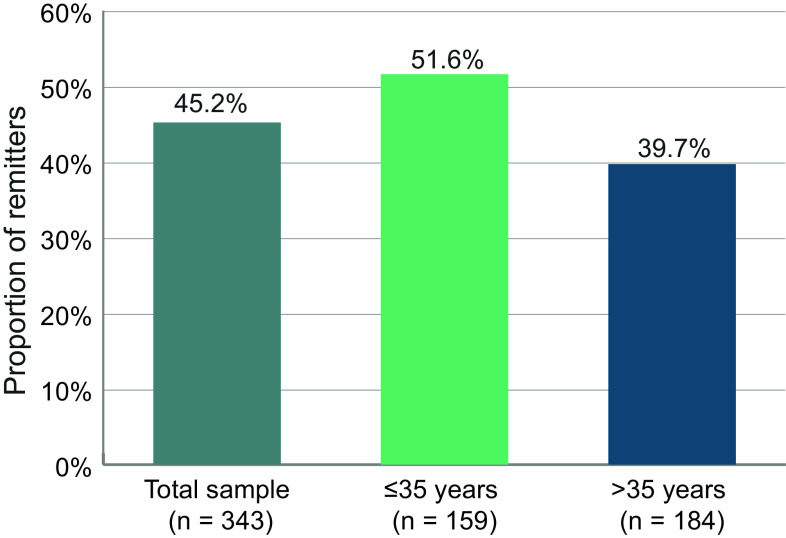
Proportion of remitters at month 6. Patients were considered remitters if they had a score of 3 or less on the following BPRS items for at least 3 months, that is, two consecutive visits: grandiosity, suspiciousness, unusual thought content, hallucinatory behavior, conceptual disorder, mannerisms, and blunted affect.

The proportion of patients who achieved remission during the first 6 months, defined as a score of 3 or less on key BPRS items for two consecutive visits, was 45.2% for the overall sample (Figure 3). A total of 51.6% of the patients ≤35 years achieved remission, but only 39.7% of the older patients did so.

### Disease severity: CGI-S

**Figure 4.**
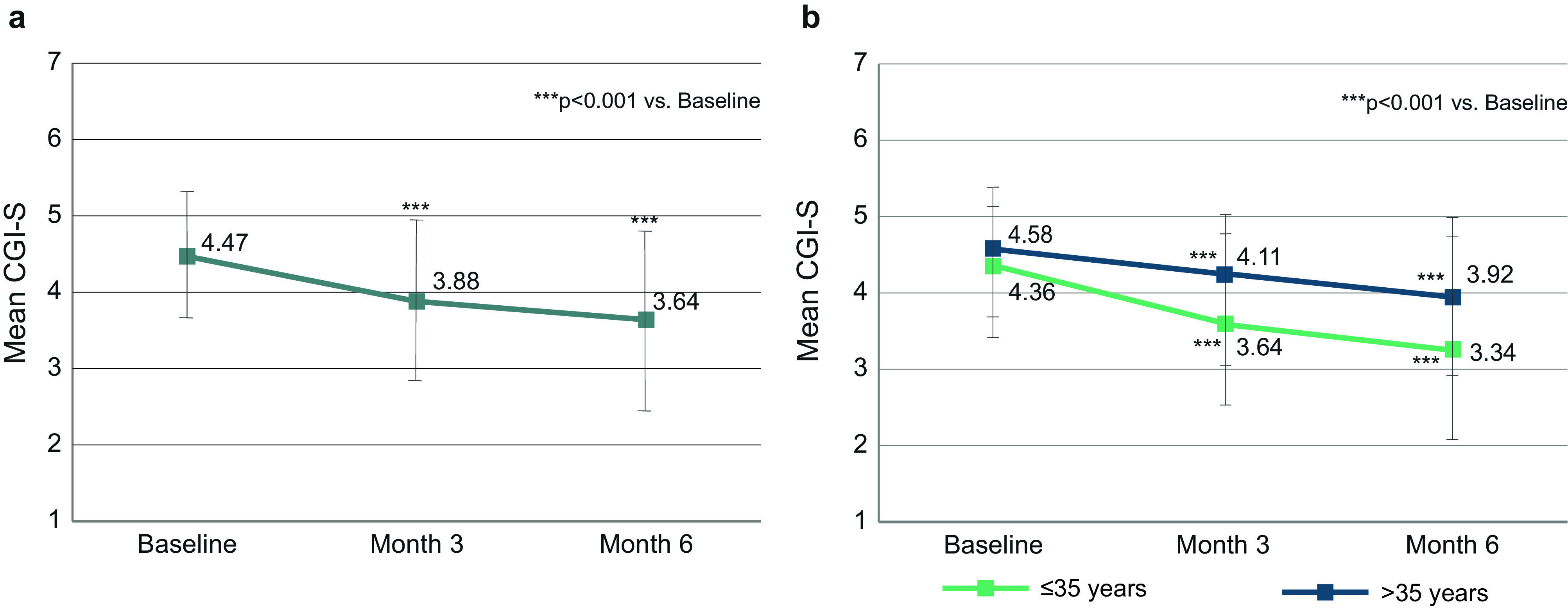
CGI. (A) Change of CGI in the total population (*n* = 395). (B) Change of CGI in patients ≤35 years (*n* = 193) and >35 years (*n* = 202). Error bars represent standard deviations. Missing data were imputed using the last observation carried forward method.

At baseline, the mean CGI-S score was 4.47 (SD 0.90) (Figure 4A). During 6 months of treatment, the mean score improved by 0.84 (SD 1.12) to reach 3.64 (SD 1.16) at month 6. Compared to baseline, improvements at month 3 and month 6 were statistically significant (*p* < 0.001), indicating a sustained effect. Younger patients ≤35 years had less disease severity at baseline, and throughout the first 6 months of treatment, compared to older patients (Figure 4B).

### CGI-S response

**Figure 5.**
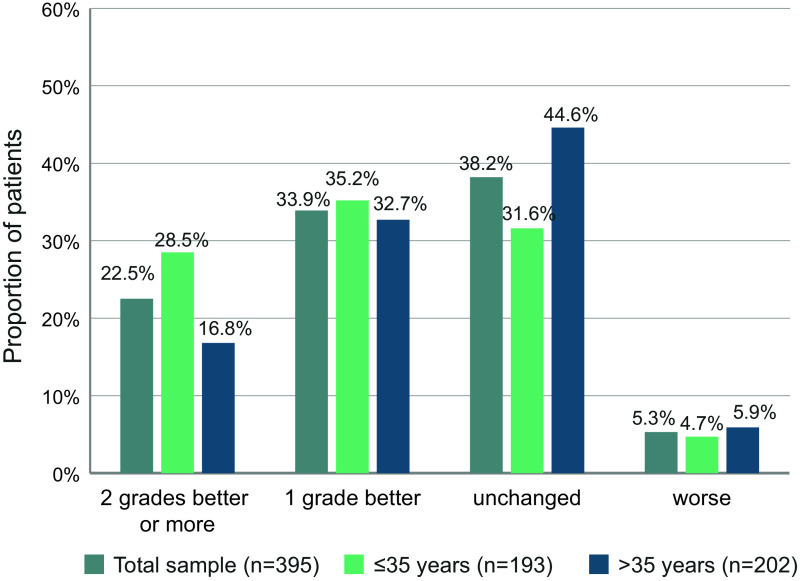
Proportion of patients with defined changes of CGI-S scores between baseline and month 6.

A total of 56.4% of the patients showed a response on the CGI-S scale, with 33.9% having a score 1 level better than baseline and 22.5% 2 levels better or more (Figure 5). For 38.2% of the patients, CGI-S did not change during the first 6 months of treatment while it deteriorated for 5.3% of the patients. The proportion of patients improving on the CGI-S was higher for the younger population ≤35 years.

### Correlation of BPRS and CGI

**Figure 6.**
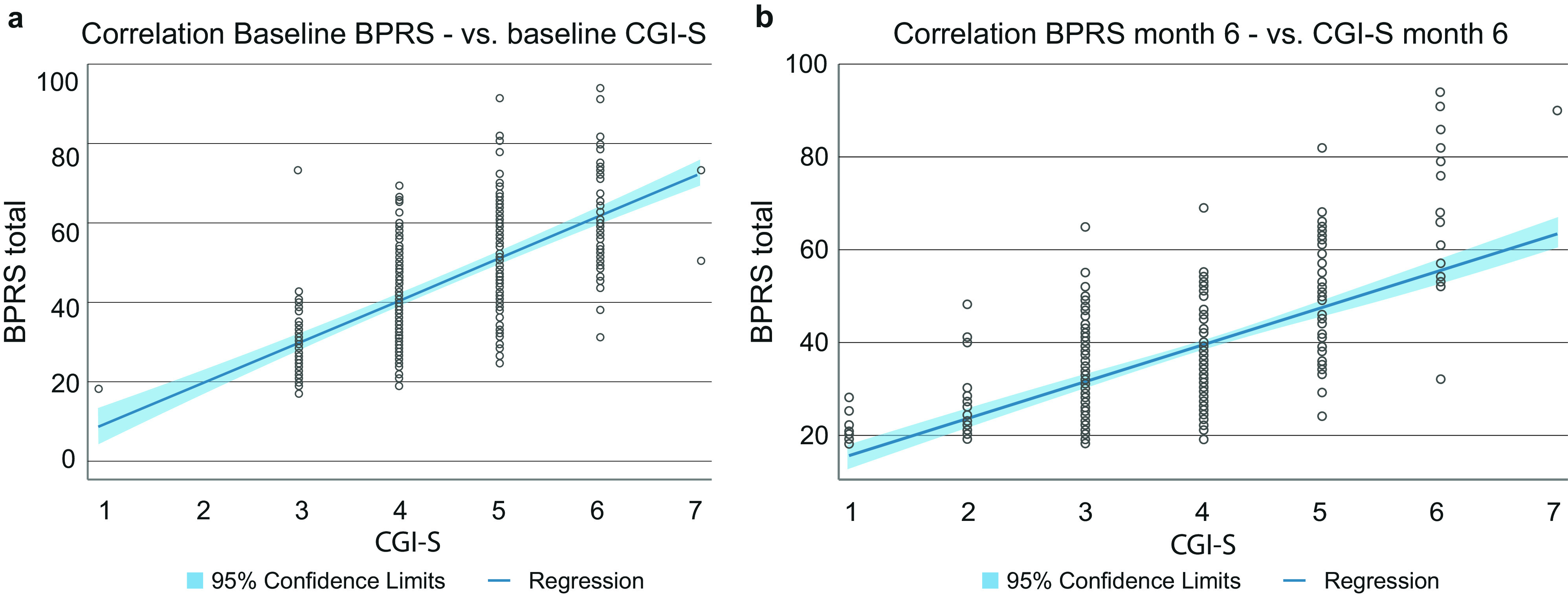
Correlation of BPRS total score and CGI-S score. (A) Correlation at baseline. Pearson correlation coefficient is 0.65. (B) Correlation at month 6. Pearson correlation coefficient is 0.67. Blue solid line shows regression, blue shaded area shows 95% confidence limits.

BPRS and CGI score were correlated with Pearson coefficients of 0.65 at baseline and 0.67 at month 6 (Figure 6).

### Composite response: BPRS and CGI

**Figure 7.**
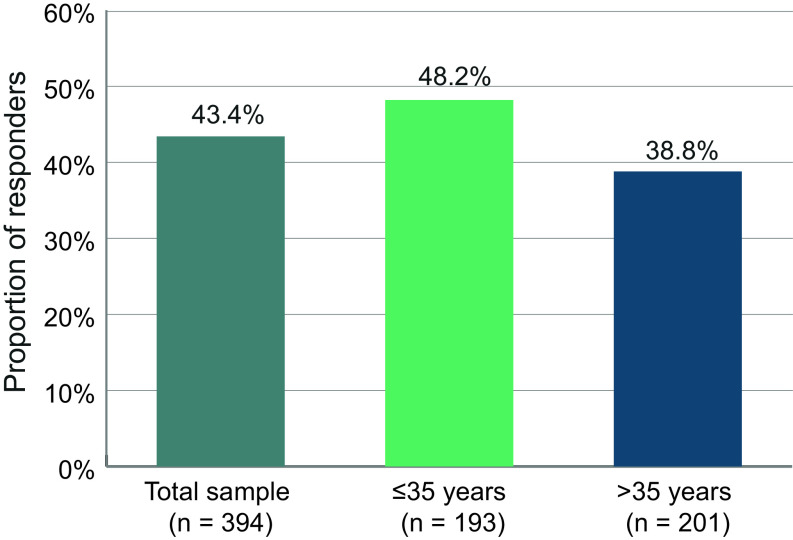
Proportion of responders on both the BPRS and CGI scales. Patients were considered responders if they showed an improvement of at least 20% of the BPRS total score and an improvement in CGI of at least one level.

Based on the correlation of BPRS and CGI over time, we examined how many patients responded on both scales. A composite response on both the BPRS and CGI scales, defined as 20% improvement on the BPRS and improvement of CGI of at least one level, was achieved by 43.4% of the patients in the total sample (Figure 7). A total of 48.2% of the younger patients ≤35 years achieved this goal, compared to only 38.8% of the older patients.

### Safety and tolerability

One-hundred and ninety-two patients (46.9%) experienced adverse events during the first 6 months of treatment. Seven patients (1.7%) discontinued because of adverse events. Adverse events that occurred in more than 1% of the patients are listed in Table 2. Akathisia, other extrapyramidal symptoms and weight gain were rare. There were many other adverse events that were very rare in frequency and each occurred in less than 1% of all patients.

**Table 2.** Adverse events that occurred in more than 1% of the patients.

	Number of patients (%), *n* = 409
Any adverse event	192 (46.9)
Psychotic symptoms	31 (7.6)
Extrapyramidal symptoms	12 (2.9)
Akathisia	8 (2.0)
Injection site pain	6 (1.5)
Weight increased	6 (1.5)

## Discussion

We studied AOM treatment in real-world samples of patients with schizophrenia who participated in two noninterventional studies in Canada and Germany [16, 17]. Although there are some differences between the samples (Canadian patients were on average younger and less severely ill at baseline), a feasibility analysis showed that pooling the data would produce valid results. Pooling the results gave us the chance to have larger subgroups of patients in our analyses.

In the present analysis, we examined whether symptoms improved, if this was clinically relevant (i.e., translated into a response of at least 20% BPRS improvement/1 level better on CGI-S), and if patients reached a state of remission.

At baseline, patients had a mean BPRS total score of 48.1, reflecting moderate to marked illness severity [20]. The improvement over 6 months was 38.5%, corresponding to a CGI-I rating between “minimally improved” and “much improved” [20]. At month 6, the mean BPRS total score was 36.5, reflecting mild to moderate illness severity [20]. Improvement in younger patients was more pronounced than in older patients, taking into account that younger patients started out with less severe symptoms initially. For stratification of the age groups, we chose a cutoff at 35 years, because patients up to 35 years showed significantly better results than patients older than 35 years in the QUALIFY study [13]. Compared to QUALIFY, our sample has a larger percentage of young patients (48.7% in this analysis vs. 29.2% in the QUALIFY AOM study arm).

For response on the BPRS, we chose an improvement of 20% as the response criterion. In acutely ill patients, a greater improvement is warranted to define adequate response [20]; however, patients in our studies were outpatients who had been pretreated (as per local label), and in the German study treating clinicians were explicitly asked if they deemed their patients stable, which was the case for 87.9% of patients. Therefore, a 20% improvement seems adequate for a response definition. A total of 54.4% of the patients analyzed here met this criterion.

For remission, we modified the criteria proposed by Andreasen et al. [21]. Specifically, we shortened the duration criterion from 6 to 3 months, given that the timeframe under study was only 6 months altogether. 45.2% of the patients achieved remission during either the first 3 months, the last 3 months or throughout the study.

CGI values in our sample decreased from a mean of 4.47 to 3.64, that is, from moderate to marked illness severity at baseline to mild to moderate illness severity at month 6, in accordance with the BPRS results. We noted a response on the CGI scale for 56.4% of patients, which is also well in line with BPRS responses. Furthermore, we noted a correlation of BPRS and CGI results that aligns well with data reported by Leucht et al. [20]. We therefore investigated how many patients had responded on both the BPRS and CGI scales, and found that this was the case for 43.4% of the patients.

Since many of the patients under study were already stabilized at baseline, one would expect that most would just remain at the same level of symptom and illness severity during the study. Nevertheless we found that more than half of the patients showed clinically relevant improvements.

Improvements in terms of symptom severity, illness severity, response, and remission appeared more pronounced in younger patients up to 35 years compared with the patients older than 35 years, suggesting that young patients may particularly benefit from AOM treatment. The argument has been raised that LAI antipsychotics should be offered early in the course of the disease [15, 22, 23], which is also reflected in the treatment guideline of Québec [8] as well as a consensus statement from the Taiwanese Society of Biological Psychiatry and Neuropsychopharmacology [24].

AOM appeared safe and tolerable in our analysis. One-hundred and eighty-four patients did not use oral aripiprazole for exactly 2 weeks after starting AOM, as was indicated in the product label when the studies were conducted. An alternative initiation regimen is now available in Europe and Canada which consists of injecting two doses of AOM at two different sites with one oral dose of 20 mg aripiprazole as supplementation, which in simulations displayed a comparable pharmacokinetic profile to the single-injection start regimen with concurrent 14-day oral administration [25]. The two-injection start regimen is expected to make such dosage excursions less likely. Akathisia, other extrapyramidal symptoms and weight gain were rare. In the Canadian study, additional adverse events occurred after month 6, explaining the higher rates of akathisia and weight gain mentioned in Mustafa et al. [17].

Owing to the design of the original studies, our study has some limitations. This is a pooled analysis, so that all analyses done here have be considered as post hoc. Many of the included patients had been treated with oral aripiprazole before switching to AOM, potentially enriching our sample with patients who tolerated aripiprazole. Patients were willing to try AOM treatment, so that there may have been some expectation bias. The patients were not blinded to treatment, and there was no control group, so that confounders cannot be excluded or identified.

LAIs have been found to be more effective in preventing relapse and hospitalization than oral medication in different research settings [9]. Specifically for AOM, this was found in the PRELAPSE trial [15]. In a recent meta-analysis of different LAIs, AOM performed particularly well against other LAIs in head-to-head comparisons regarding acceptability [26] and was recommended in the network meta-analysis along with the 3-month formulation of paliperidone, the 1-month formulation of paliperidone and olanzapine as first-line maintenance treatment options in patients with schizophrenia and related nonaffective psychotic disorders.

In the PRELAPSE trial, staff at centers randomized to AOM treatment were trained in communication skills to help them discuss AOM treatment with their patients and make a shared decision [15]. It is very likely that communication plays a major role in motivating patients to adhere to their treatment, thus realizing its full potential.

We found that patients with schizophrenia treated under real-world conditions experienced symptomatic improvements after starting AOM, even though the patients were pretreated (as per local label) and many of them were already symptomatically stable. Therefore, our findings support the notion that treatment with AOM can not only prevent relapses and hospitalizations, but can also lead to further improvements in pretreated and stabilized patients with schizophrenia.

## Conclusion

We present evidence for the effectiveness and safety of AOM for patients with schizophrenia under real-world conditions. We found that patients experienced symptomatic improvements during 6 months of AOM use, even though many of the patients were stable and pretreated (as per local label). The treatment appeared tolerable and safe.

## Data Availability

The data that support the findings of this study are available from the corresponding author, D.S., upon reasonable request.
